# Geocoding rural addresses in a community contaminated by PFOA: a comparison of methods

**DOI:** 10.1186/1476-069X-9-18

**Published:** 2010-04-21

**Authors:** Verónica M Vieira, Gregory J Howard, Lisa G Gallagher, Tony Fletcher

**Affiliations:** 1 Department of Environmental Health, Boston University School of Public Health, 715 Albany Street Talbot 4W, Boston, MA 02116, USA; 2 Department of Environmental Studies, Dickinson College, Kaufman Building Room 131, Carlisle, PA 17013, USA; 3 London School of Hygiene and Tropical Medicine Public Health and Environmental Research Unit, Keppel Street, London WC1E 7HT, UK

## Abstract

**Background:**

Location is often an important component of exposure assessment, and positional errors in geocoding may result in exposure misclassification. In rural areas, successful geocoding to a street address is limited by rural route boxes. Communities have assigned physical street addresses to rural route boxes as part of E911 readdressing projects for improved emergency response. Our study compared automated and E911 methods for recovering and geocoding valid street addresses and assessed the impact of positional errors on exposure classification.

**Methods:**

The current study is a secondary analysis of existing data that included 135 addresses self-reported by participants of a rural community study who were exposed via public drinking water to perfluorooctanoate (PFOA) released from a DuPont facility in Parkersburg, West Virginia. We converted pre-E911 to post-E911 addresses using two methods: automated ZP4 address-correction software with the U.S. Postal Service LACS database and E911 data provided by Wood County, West Virginia. Addresses were geocoded using TeleAtlas, an online commercial service, and ArcView with StreetMap Premium North America NAVTEQ 2008 enhanced street dataset. We calculated positional errors using GPS measurements collected at each address and assessed exposure based on geocoded location in relation to public water pipes.

**Results:**

The county E911 data converted 89% of the eligible addresses compared to 35% by ZP4 LACS. ArcView/NAVTEQ geocoded more addresses (n = 130) and with smaller median distance between geocodes and GPS coordinates (39 meters) than TeleAtlas (n = 85, 188 meters). Without E911 address conversion, 25% of the geocodes would have been more than 1000 meters from the true location. Positional errors in TeleAtlas geocoding resulted in exposure misclassification of seven addresses whereas ArcView/NAVTEQ methods did not misclassify any addresses.

**Conclusions:**

Although the study was limited by small numbers, our results suggest that the use of county E911 data in rural areas increases the rate of successful geocoding. Furthermore, positional accuracy of rural addresses in the study area appears to vary by geocoding method. In a large epidemiological study investigating the health effects of PFOA-contaminated public drinking water, this could potentially result in exposure misclassification if addresses are incorrectly geocoded to a street segment not serviced by public water.

## Background

In rural areas, residential addresses have historically been rural routes used by mail carriers and not indicative of the physical location of a residence. As a result, street-level geocoding match rates are lower in rural areas compared to urban areas [1-4]. One opportunity for recovering valid street addresses is county-level E911 readdressing projects. With the goal of improving emergency response time, these projects assign new street addresses to all residents that were previously only identified by rural route boxes. This often results in renumbering or renaming of existing street addresses to accommodate the influx of new street assignments in certain areas. Data obtained from the E911 administrators therefore provide pre-E911 addresses (including rural route boxes as well as street addresses) and their corresponding post-E911 valid street addresses. Another method for converting rural routes to street addresses uses data from the U.S. Postal Service (USPS) that is updated every month. The standard address-correction software package, ZP4, includes a LACS (locatable address conversion system) database from USPS that quickly converts rural routes and pre-E911 street addresses to valid post-E911 street addresses. An additional concern for rural areas is that successful geocoding of these newly converted addresses is limited by the accuracy of street map files and their ability to keep pace with E911 readdressing projects.

Geographic location is an important aspect of assessing environmental exposures for residents in epidemiological studies. As part of the exposure assessment for community health studies investigating the potential health effects of perfluorooctanoate (PFOA, C8), addresses of residents living in the mid-Ohio River Valley will be geocoded http://www.c8sciencepanel.org/index.html. PFOA was released from the DuPont Washington Works facility in Parkersburg, West Virginia and subsequently detected in public drinking water districts in Ohio and West Virginia during well survey sampling in 2002 [5]. A class action lawsuit brought by the surrounding communities against DuPont resulted in a settlement agreement whereby Brookmar, Inc., an independent company, conducted a year-long cross-sectional survey (August 2005 - August 2006) called the C8 Health Project [5,6]. Approximately 69,000 individuals who have lived in at least one of six affected water districts near the DuPont Washington Works Plant provided a blood sample and completed a questionnaire regarding residential and occupational history, water use, health history, and demographic information. The settlement also established a Science Panel of public health scientists to assess whether or not there is a probable link between PFOA exposure and disease in the community.

Errors in geocoding could result in exposure misclassification and bias study results [7-10]. Positional errors may be due to inaccurate street reference files used in geocoding and default offsets that do not reflect the true distance of homes from the street centerline [11,12]. To determine the most accurate method for geocoding, and thereby reduce error in the exposure assessment for the C8 Science Panel studies, we conducted a secondary analysis of existing data using GPS measurements and self-reported addresses to compare the LACS database in ZP4 to E911 readdressing project data for converting pre-E911 rural routes and street addresses to valid post-E911 street addresses. We also compared TeleAtlas and ESRI ArcView tools for geocoding both pre- and post-E911 conversion addresses. We were interested in the number of addresses successfully converted and geocoded as well as the accuracy of the geocoded locations. We calculated the positional errors, or the distance between the geocoded address and the "true" location measured using a global positioning system (GPS). The objectives of this paper were to recover valid street addresses for rural routes and pre-E911 street addresses and determine the accuracy of street files used for geocoding the recovered addresses. Geocoded locations were mapped with a geographic information system (GIS) to assess the spatial distribution of positional errors and the impact on exposure assessment.

## Methods

### Study Residences

The 135 addresses available for this analysis were self-reported by participants in the C8 Health Project who were later recruited in 2007 for an ongoing study of PFOA toxicokinetics [13]. Participants were all residents of three towns in Wood County, West Virginia located near the Ohio River who were exposed to PFOA via public drinking water serviced by the local water district. At the time participants were asked to complete the C8 Health Project questionnaires, Wood County was undertaking an E911 readdressing project, so the self-reported addresses included pre- and post-E911 converted addresses. As part of the PFOA toxicokinetic study, five home visits per participant had been completed. GPS measurements (longitude and latitude) were obtained in front of participants' homes using a GARMIN eTrex (Garmin International, Inc., Olathe, KS), within 9.1 meters (30 feet) of the front door. Participants were asked about the E911 status of their self-reported address and address information was updated if an E911 conversion occurred or if the participant moved between home visits. A constraint of the PFOA toxicokinetic study was that participants were no longer included if they moved outside of the three towns. Participants provided informed consent for the GPS measurements and the geocoding of their addresses. To protect confidentiality, we did not map geocoded addresses.

### Geocoding

Prior to any converting or geocoding, addresses were cleaned and standardized using ZP4 address correction software without the LACS database (version expiring April 1 2009; http://www.semaphorecorp.com). We converted rural route boxes and old street addresses using (1) the automated ZP4 LACS database and (2) a data table supplied by Wood County and described as comprehensive but likely incomplete. The table included over 12,000 pre-E911 addresses and their corresponding post-E911 conversion addresses for 24 towns in Wood County, with approximately 6,500 rural route boxes, 5,000 street addresses that were renumbered or renamed, and 500 Post Office (PO) Boxes. We also cleaned and standardized the addresses in the E911 table using the ZP4 address correction engine, and then performed case-insensitive string matching to match self-reported addresses to pre-E911 addresses for conversion.

The geocoding was performed using two methods. We submitted the addresses to the EZLocate internet-based geocoding client from TeleAtlas (v2.47; http://www.geocode.com) for batch geocoding using the USA_Geo_002 database, their most current street data. We also geocoded the data in-house with the geocoding tools in ESRI ArcView version 9.3 (Redlands, CA) using the ESRI StreetMap Premium North America NAVTEQ 2008 enhanced street dataset as the reference address locator. Addresses were matched using a spelling sensitivity of 70 and a minimum match score of 65.

### Data Analysis

Because some participants moved between home visits, there were 1 to 5 GPS readings for each self-reported address. If there was more than one GPS measurement taken at the self-reported address, we used an averaged measurement in our analysis. The GPS measurements represent the "true" locations, so we confirmed the accuracy of the averaged coordinates by comparing the longitude and latitude measurements obtained from the online Wood County GIS database http://www.onlinegis.net/WvWood/, which is a product of the E911 readdressing project. We also used the online database to verify the E911 status of the self-reported addresses. GPS measurements and public water pipes were mapped with GIS to confirm all addresses were exposed.

Self-reported addresses were first geocoded using TeleAtlas and ArcView/NAVTEQ prior to any conversion. Eligible addresses were then converted using the ZP4 LACS module and county E911 data table. The post-E911 converted addresses were again geocoded using the two geocoding methods. We converted all measurements of longitude and latitude to meters using the North America Datum 1983 State Plane West Virginia North FIPS 4701 projection. Positional error was calculated as the difference in meters between the GPS measurements and the geocoded results. We compared the geocoding match rate for TeleAtlas and ArcView/NAVTEQ before and after ZP4 LACS and E911 table conversions and report the median, 25th and 75^th ^percentile of the positional error distributions in meters [14].

Geocoded addresses are an important component to exposure assessment in the C8 Science Panel studies. To investigate the affects of positional error on exposure status, we mapped geocoded locations and the public water distribution system to assess whether addresses would be classified as exposed. We also examined the spatial distribution of positional errors to determine if certain streets were more difficult to geocode or if errors were widespread. The Institutional Review Board of Boston University Medical Center approved this research.

## Results

Of the 135 addresses, 71 (53%) were self-reported with a valid post-E911 street address that we confirmed using the online Wood County GIS database. These street addresses were not included for conversion with the ZP4 LACS and county E911data table. The remaining self-reported addresses included 37 pre-E911 street addresses (27%), 26 rural route boxes (19%), and 1 PO Box (1%), for a total of 64 potential address conversions.

When we compared the two methods for address conversions, the E911 table was more successful in recovering valid street addresses. The E911 table converted 32 of the 37 street addresses (86%) and 24 of the 26 rural route boxes (92%). The LACS database in ZP4 converted 13 of the 37 street addresses (35%) and 9 of the 26 rural route boxes (35%). The 9 rural routes converted by ZP4 LACS were also converted by the E911 table, and the resulting post-E911 conversion addresses were the same for both methods. Of the 13 street addresses converted using ZP4 LACS, 10 were converted by the E911 table, again to the same post-E911 conversion address. By running ZP4 LACS after matching to the E911 table, we converted 3 more addresses for a total of 59 converted addresses, and improved the conversion rate from 88% to 92% with a minimal amount of additional effort. The PO Box was not converted by either method.

Having determined that the E911 table was more effective for converting addresses, we then compared geocoding results using TeleAtlas and ArcView/NAVTEQ. We were interested in whether geocoding results were affected by the E911 readdressing project, so the 135 self-reported addresses were first geocoded prior to conversion (Table 1). As expected, the rural routes boxes and PO Box were not geocoded by either method. Of the 37 self-reported pre-E911 street addresses, TeleAtlas geocoded 34 compared to 29 geocoded by ArcView/NAVTEQ, although the positional errors were similar for both methods. For the 71 self-reported post-E911 street addresses, ArcView/NAVTEQ geocodes were more accurate than TeleAtlas with a median positional error 3 times smaller and almost all of these addresses geocoded (70 of 71 compared to 52 of 71).

**Table 1:** Geocoding Results of Self-reported Addresses before E911 Conversion.

		TeleAtlas		ArcView/NAVTEQ	
		
Address status	N	**Error**^**1**^	**N (%)**^**2**^	**Error**^**1**^	**N (%)**^**2**^
Self-reported Post-E911 Street	71	132 (75, 309)	52 (73%)	40 (17, 70)	70 (99%)
Self-reported Pre-E911 Street	37	243 (117, 418)	34 (92%)	236 (153, 413)	29 (78%)
Total Addresses^3^	135	174 (81, 357)	86 (64%)	62 (25, 151)	99 (73%)

^1^Positional errors are presented as median distance (25^th^, 75^th ^percentile) in meters between geocoded location and GPS measurements.

^2^Represents the number of successfully geocoded addresses and the percent (%).

^3 ^The 26 rural route boxes and the 1 PO Box were not geocoded by either method.

We then geocoded the 59 converted post-E911 street addresses and compared results for TeleAtlas and ArcView/NAVTEQ (Table 2). Again, ArcView/NAVTEQ geocoded the addresses with more accuracy and success, with all 59 addresses geocoded and positional errors on average an order of magnitude less than TeleAtlas. When compared to the GPS measurements, 18 of the 24 converted rural route boxes (75%) and 29 of the 35 street addresses (83%) were within 100 meters and all were within 200 meters. TeleAtlas geocoded 31 of the 59 converted addresses (52%); 7 (23%) were within 100 meters and 10 (32%) were within 200 meters the GPS measurement.

**Table 2:** Geocoding Results after E911 Conversion.

		TeleAtlas		ArcView/NAVTEQ	
		
Address status	N	**Error**^**1**^	**N (%)**^**2**^	**Error**^**1**^	**N (%)**^**2**^
Converted Pre-E911 Street	35	373 (204, 629)	20 (57%)	20 (15, 58)	35 (100%)
Converted Rural Route Boxes	24	280 (80, 2362)	11 (46%)	60 (24, 200)	24 (100%)
Total Addresses^3^	135	188 (77, 434)	85 (63%)	39 (16, 73)	130 (96%)

^1^Positional errors are presented as median distance (25^th^, 75^th ^percentile) in meters between geocoded location and GPS measurements.

^2^Represents the number of successfully geocoded addresses and the percent (%).

^3^Includes the results for the 71 self-reported post-E911 street addresses and the 2 self-reported pre-E911 street addresses that were not converted. The 1 PO Box was not geocoded by either method.

E911 conversion increased the number of addresses geocoded by ArcView/NAVTEQ from 99 to 130 and improved the positional accuracy (median distance (25th, 75^th ^percentile) of 62 (3, 151) meters compared to 39 (16, 73 meters). Of the 130 addresses, 109 (84%) were within 100 meters and 128 (98%) were within 200 meters of the GPS measurement. Converting the addresses did not improve the number of addresses geocoded by TeleAtlas or the positional accuracy. A total of 85 addresses were geocoded by TeleAtlas after E911 conversion compared to 86 prior to conversion. Of the 85 addresses, 28 (33%) were within 100 meters and 44 (52%) were within 200 meters of the GPS measurement.

From among the 135 self-reported addresses, the E911 readdressing project resulted in the renumbering and renaming of 37 street addresses, 35 of which were successfully converted (95%). We compared the geocoding results of these 35 street addresses before and after conversion and found that TeleAtlas and ArcView/NAVTEQ successfully geocoded 32 and 28 addresses prior to conversion and 20 and 35 addresses after conversion, respectively. There were 16 addresses that ArcView/NAVTEQ and TeleAtlas both successfully geocoded before and after conversion. Comparing TeleAtlas and ArcView/NAVTEQ, the median distance of the positional errors (25^th ^and 75^th ^percentiles) for these 16 addresses were 350 (160, 888) meters and 205 (100, 578) meters before conversion and 419 (252, 749) meters and 19 (15, 53) meters after conversion, respectively. ArcView/NAVTEQ clearly performed better than TeleAtlas, especially after converting the addresses with the E911 data and ZP4 LACS. When we examined the positional accuracy of street addresses that were geocoded using ArcView/NAVTEQ but without E911 conversions, 12 of the 16 addresses (75%) were more than 100 meters from the GPS measurement, and 4 (25%) were geocoded more than 1000 meters from the true location.

To determine what impact positional errors may have on exposure assessment, we mapped the geocoded addresses and compared their locations to the distribution of public water pipes in the study area. Participants located on piped public water were considered exposed. Maps are not shown to protect the confidentiality of participants. Despite the positional errors observed for the geocoded self-reported addresses prior to E911 address conversion, only 7 of the 86 TeleAtlas geocodes (8%) and 1 of the 99 ArcView/NAVTEQ geocodes (1%) were incorrectly classified as unexposed. This is due to the fact that many of the self-reported addresses that were inaccurately geocoded were still geocoded to the correct street, albeit kilometers away, and the entire length of the street happened to be on public water. When we examine addresses that were converted using county E911 data and ZP4 LACS, all ArcView/NAVTEQ geocodes were correctly classified as exposed. However, TeleAtlas had more difficulty in geocoding the addresses accurately, which resulted in the exposure misclassification of 5 out of 31 (16%) addresses. We examined the location of geocoded addresses with highest positional errors and saw they were evenly distributed throughout the study area.

## Discussion

The current study is a secondary analysis of existing data with the goal of informing geocoding decisions for a large epidemiological investigation of the health effects of PFOA in a rural community. A strength of this study was the availability of GPS measurements, but a related weakness was that the existing data only included 135 rural addresses in Wood County, WV. Bearing in mind the small numbers, our analysis showed that the county E911 data table appears to perform better than the LACS database in ZP4, substantially converting more self-reported addresses. Although the E911 table did not include 7 of the self-reported pre-E911 addresses, the table did include other street addresses on the same street, so while the table may be incomplete, it is not missing entire streets. LACS ZP4 was able to convert 3 of the 7 addresses missed by the E911 table. Because ZP4 is already used to clean and standardize addresses, we recommend also running the LACS database. While the return of additional addresses may be small, the amount of additional work is negligible.

We also determined that ArcView with the ESRI StreetMap Premium North America NAVTEQ 2008 enhanced street dataset geocoded more addresses and with better accuracy then the TeleAtlas EZ-Locate internet-based geocoding service. TeleAtlas geocoded more of the pre-conversion street addresses, suggesting that their street files for this area in West Virginia are not as current as the NAVTEQ StreetMap. After E911-converting the addresses, ArcView/NAVTEQ was able to geocode 96% of the addresses with a median positional error of only 39 meters. In terms of exposure misclassification, we showed that despite the large geocoding errors, most addresses were accurately classified as exposed. This is mainly due to the fact that inaccurate geocodes consisted primarily of renumbered street addresses, so an address geocoded to any location on that street was still correctly classified as exposed if the entire length of street was serviced by public water. An additional component to our exposure assessment which we do not address in this comparison is that years of pipe installation, which determines exposure period, varies by street segment. Therefore, geocoding errors may still impact exposure values even if addresses are correctly classified to a water district. Because water distribution systems tend to grow over time with more recent pipe typically installed further from a population center, we were interested in knowing whether errors were geographically concentrated in a particular region. Streets affected by E911 renumbering, with errors typically greater than 0.5 kilometers in this analysis, were evenly distributed in our study area. Therefore, the entire water distribution system is equally affected by the E911 readdressing issues, and geocoding errors are just as likely to occur to those on older pipe segments as those on newer pipe segments.

Improving the accuracy of geocoded addresses reduces uncertainty and bias in studies of geographically-based environmental exposures, but can often be costly and labor intensive [15,16]. ZP4 with the LACS database is automated, cost-efficient, and converts addresses in a few minutes, but the LACS database as not as current as the Wood County E911 data. Although the E911 table was effective in reducing positional errors, it required some manual interaction in matching, and completeness is difficult to determine. E911 data is also unavailable in certain counties, either because readdressing was completed so long ago that E911 conversion tables no longer exist or E911 readdressing has yet to occur. Other E911 programs have commercial mapping companies provide directions for emergency personnel so data are privately owned. Nonetheless, we strongly recommend contacting county officials to inquire about the availability of E911 data when geocoding rural addresses.

One of the few examples evaluating E911 data for geocoding can be found in simulation studies for all rural addresses in Carroll County, Iowa on the predictability of positional errors and potential impact on health outcome studies [17,18]. Rural addresses were geocoded with an automated method using TIGER street files, E911 data, and an aerial photo as the gold standard. The median positional error of the E911 geocoded addresses was 46 meters and 211 meters for the automated method [18].

Even if addresses are successfully converted, reference datasets (street files, parcel records) must be updated concurrently for accurate geocoding to occur. In our analysis, geocoding of post-E911 converted addresses with ArcView using NAVTEQ street files was more accurate than geocoding by a commercial service (TeleAtlas), presumably due to the incorporation of more E911 address data in the NAVTEQ files. In addition, median positional errors using ArcView/NAVTEQ were smaller for post-E911 converted street addresses (20 meters, Table 2) compared to pre-E911 converted street addresses (236 meters, Table 1). Conversely, pre-E911 converted street addresses geocoded by TeleAtlas had a smaller median positional error (243 meters, Table 1) than post-E911 converted street addresses (373 meters, Table 2). A possible reason for this difference is that TeleAtlas reference street files may include more pre-E911 street information. This is further supported by the fact that TeleAtlas geocoded only 20 of 35 post-E911 converted street addresses compared to 34 of 37 pre-E911 converted street addresses.

A study by Ward et al. [19] examined accuracy in geocoding residential addresses in rural areas with ArcView using U.S. Census Bureau TIGER 2000 street map files, a common and freely-available street reference file. Geographic locations were determined by global positioning system (GPS) measurements at homes and geocoded with ArcView/TIGER 2000 and an automated process by a commercial firm. In rural areas, they found 56% of those geocoded with ArcView/TIGER 2000 and 28% of those geocoded by the commercial firm were within 100 meters of the GPS measurement. These percentages were smaller than addresses located in towns (ArcView/TIGER 2000, 81%; commercial firm, 84%). Interpolation issues due to longer street segments and greater distances between houses and street centerlines in rural areas contribute to positional errors in geocoding. The exposure in this study was proximity to crop fields, and geocoding errors affected classification of homes at the 100 meter distance [19].

Another challenge in geocoding addresses both in rural and urban areas is PO Boxes. While the Wood County E911 table in this study included some PO Boxes, they are not usually affected by E911 readdressing programs and, therefore, not consistently found in E911 tables. A study of breast cancer in California described challenges of post office box addresses in geocoding and determined an alternative address using tracing methods [20]. Street addresses were collected from U.S. Postmaster post office box rental records, but only yielded results for 34% of post office box addresses and required comparison of subject names and dates of interest. A delivery-weighted zip code centroid was also assigned to the post office box. When compared to the street address, 25% were more than 4.3 miles away from the centroid.

## Conclusions

Despite the limitations of this study, our results suggest that the Wood County E911 readdressing data were very useful for improving geocoding success rates, and we recommend using county E911 data in combination with the ZP4 LACS database. Furthermore, ArcView with NAVTEQ street maps geocoded addresses in this rural study area with higher accuracy than TeleAtlas. We also showed that converting rural routes alone is not sufficient for accurate geocoding. Because of the renumbering and renaming of street addresses that occurred as a result of the E911 readdressing projects, street addresses should be compared to county E911 data for potential conversion as well. Although exposure to contaminated public drinking water among the participants in this analysis was not greatly affected by positional errors, E911 data was important for maximizing the number of addresses geocoded and making exposure assessments possible.

## Abbreviations

GIS: Geographic Information Systems; GPS: Global Positioning System; E911: Enhanced 911; LACS: Locatable Address Conversion System; PFOA, C8: Perfluorooctanoate; PO: Post Office; USPS: United States Postal Service

## Competing interests

The authors declare that they have no competing interests.

## Authors' contributions

VV conceived of the study, carried out the data analysis, and drafted the manuscript. GH conducted the geocoding and GPS validation, and collaborated on all analytical and editorial decisions. LG contributed to the literature review and provided editorial support. TF assisted in the analysis and editing. All authors read and approved the final manuscript.
